# Bioengineering liver microtissues for modeling non-alcoholic fatty liver disease

**DOI:** 10.17179/excli2022-5892

**Published:** 2023-03-20

**Authors:** Negar Aasadollahei, Niloufar Rezaei, Reihaneh Golroo, Tarun Agarwal, Massoud Vosough, Abbas Piryaei

**Affiliations:** 1 Department of Regenerative Medicine, Cell Science Research Center, Royan Institute for Stem Cell Biology and Technology, ACECR, Tehran, Iran; 2 Department of Developmental Biology, University of Science and Culture, Tehran, Iran; 3 Department of Bio-Technology, Koneru Lakshmaiah Education Foundation, Vaddeswaram, AP, India; 4 Experimental Cancer Medicine, Institution for Laboratory Medicine, Karolinska Institute, Huddinge, Sweden; 5 Department of Biology and Anatomical Sciences, School of Medicine, Shahid Beheshti University of Medical Sciences, Tehran, Iran; 6 Department of Tissue Engineering and Applied Cell Sciences, School of Advanced Technologies in Medicine, Shahid Beheshti University of Medical Sciences, Tehran, Iran

**Keywords:** non-alcoholic fatty liver disease, in vitro modeling, bioengineering, liver microtissue, liver organoid, organ-on-a-chip

## Abstract

Non-alcoholic fatty liver disease (NAFLD) has become the world's most common chronic liver disease. However, due to the lack of reliable *in vitro* NAFLD models, drug development studies have faced many limitations, and there is no food and drug administration-approved medicine for NAFLD treatment. A functional biomimetic *in vitro* human liver model requires an optimized natural microenvironment using appropriate cellular composition, to provide constructive cell-cell interactions, and niche-specific bio-molecules to supply crucial cues as cell-matrix interplay. Such a suitable liver model could employ appropriate and desired biochemical, mechanical, and physical properties similar to native tissue. Moreover, bioengineered three-dimensional tissues, specially microtissues and organoids, and more recently using infusion-based cultivation systems such as microfluidics can mimic natural tissue conditions and facilitate the exchange of nutrients and soluble factors to improve physiological function in the *in vitro* generated constructs. This review highlights the key players involved in NAFLD initiation and progression and discussed the available cells and matrices for *in vitro* NAFLD modeling. The strategies for optimizing the liver microenvironment to generate a powerful and biomimetic *in vitro* NAFLD model were described as well. Finally, the current challenges and future perospective for promotion in this subject were discussed.

## Introduction

Non-alcoholic fatty liver disease (NAFLD) is currently one of the most common chronic liver diseases. The global prevalence of NAFLD is almost 25 %, with the highest prevalence in South America and the Middle East, and the lowest in Africa (Filozof et al., 2015[32]; Younossi et al., 2016[141]). Basic and preclinical studies are critical to improving our knowledge regarding the pathophysiology of the disease and discovering efficient medications for therapy. Due to the complexity of liver tissue and the pathophysiology of NAFLD, engineering an efficient *in vitro* NAFLD model requires the integration of several disciplines. 

Understanding the role of different cell types and their co-culture and the importance of extracellular matrix (ECM) components in the progression of diseases is substantial. The complexity of the cellular models used for NAFLD modeling ranges from two-dimensional (2D) cell cultures to simple three-dimensional (3D) cell cultures such as cell aggregates or spheroids. More complex 3D models were developed by co-culturing parenchymal and non-parenchymal (NPC) liver cells in combination with appropriate biomaterials for better-mimicking liver physiology and pathophysiology of diseases (Takebe et al., 2013[113]; Ma et al., 2016[70]; Baze et al., 2018[7]; Jin et al., 2018[54]; Zahmatkesh et al., 2022[143]). It has been demonstrated that recent *in vitro* models launched as "microtissues" or "organoids", have optimized the *in vitro* microenvironment and dramatically improved hepatic functions of the constructs, particularly when incorporated with microfluidic systems (Parmentier et al., 2018[81]; Sun et al., 2019[110]). 

This review first summarized the pathophysiological mechanisms of initiation and progression of NAFLD and described the available cell sources for *in vitro* NAFLD models. Then different strategies for optimizing the liver microenvironment and culture conditions to improve the 3D architecture and functionality of the liver equivalent constructs for the *in vitro* modeling of NAFLD were discussed. Finally, the most recent advances in NAFLD models were highlighted which provide the basis for further NAFLD research. 

## NAFLD Description and Nomenclatures

NAFLD is described as a set of heterogeneous pathological conditions with intracellular lipid accumulation in the lack of secondary causes of liver steatosis, such as excessive alcohol intake, viral hepatitis, or other liver steatosis causes. In other words, NAFLD is defined when all other causes of fatty liver disease are excluded (Chalasani et al., 2018[11]). However, this definition is not perfect because the fatty liver may coexist with the mild to moderate consumption of alcohol in patients, viral hepatitis, or metabolic dysfunction. Therefore, the negative diagnosis criteria are inappropriate and confuse clinicians in the diagnosis of NAFLD regarding the presence of other possible causes of liver steatosis. In 2020, an international consensus proposed a new nomenclature from NAFLD to metabolic dysfunction-associated fatty liver disease (MAFLD). MAFLD is defined as liver intracellular lipid accumulation in the presence of at least one of the three conditions: obesity, type 2 diabetes mellitus (T2DM), or combined metabolic disorders (Eslam et al., 2020[29]). MAFLD better describes the pathophysiology of fatty liver disease, and its diagnosis does not require excluding alcohol consumption or other chronic liver disorder. To date, the concept of MAFLD has been accepted by many experts, although for some experts, the change in nomenclature from NAFLD to MAFLD seems premature, and there are few published declarations of MAFLD (Fouad et al., 2021[33]).

## Cellular and Molecular Pathophysiology of NAFLD Initiation and Progression

**Figure 1:**
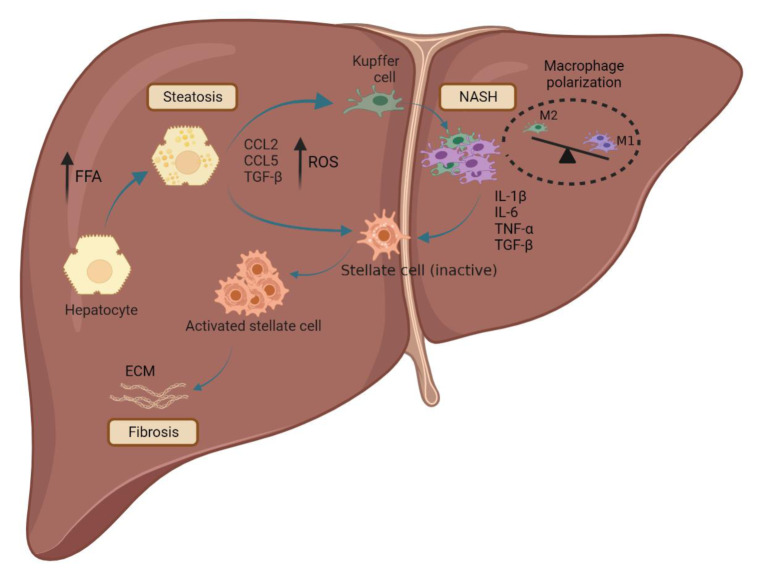
Schematic representation of NAFLD initiation and progression. Lipid overloading in hepatocytes induces hepatic steatosis which is identified by lipid accumulation. The accumulation of the lipid in hepatocytes caused the generation of oxygen-reactive species (ROS), CCL2, CCL5, and TGF-β that lead to Kupffer cell transformation from M2 to M1 phenotype, leading to inflammation and progression of the disease to the NASH. Consequently, the M1 Kupffer cells secrete pro-inflammatory cytokines, including IL-1β, IL-6, TNF-α, and TGF-β resulting in hepatic stellate cell (HSCs) activation. Finally, the activated stellate cells produce collagen, leading to the disease's progression to liver fibrosis and cirrhosis.

The term NAFLD refers to a broad spectrum of the liver diseases, from simple steatosis to non-alcoholic steatohepatitis (NASH), liver fibrosis, and cirrhosis (Ratziu et al., 2010[90]). Steatosis (simple fatty liver) is identified by the accumulation of lipids of more than 5% of the liver's total weight without any cue of hepatocellular injury. More accumulation of lipids due to mitochondrial dysfunction and generation of oxygen-reactive species (ROS), CCL2, CCL5, and TGF-β that induces Kupffer cell differentiation into pro-inflammatory M1 phenotype and progression of the disease to the NASH stage. The activated M1 Kupffer cells secrete pro-inflammatory cytokines such as IL-1β, IL-6, TNF-α, and TGF-β, leading to hepatic stellate cell (HSC) activation. The activated HSC by collagen secreting leads to the progression of the chronic disease to liver fibrosis and then cirrhosis (Figure 1(Fig. 1)) (Tsuchida et al., 2017[120]). 

Recognition of the sequence of key events that leads to a particular disease will facilitate identifying relevant therapeutic targets. Ghallab and colleagues (2021[35]), to define the sequence of events in NAFLD, were performed a multiscale time-resolved analysis in Western diet fed mice over 48 weeks and compared the results to human disease. The study determined eight key events sequence from steatosis to hepatocellular carcinoma (HCC) formation including, 1) lipid droplets accumulation, 2) lobular inflammation, 3) lipogranulomas (also called macrophage crowns), 4) reorganization of lobular zones, 5) hepatocyte death and compensatory proliferation, 6) ductular reaction, 7) liver fibrosis, and 8) HCC. Considering transcriptomic landscape, they demonstrated almost 30 percent of the genes altered in the patient suffering from NAFLD are also similarly changed in the Western diet fed mice at 12^th^ week or later. Moreover, an increasing overlap with genes altered in human HCC occurred in the mouse model at weeks 30-48. Therefore, they suggested the sequence of events recapitulates many features of NAFLD in human and could be considered as a basis for the identification of therapeutic targets. Accordingly, interventions in preceding events could ameliorate the subsequent issue successfully, e.g., antagonizing the inflammation ameliorates fibrosis progression.

It is established that ECM, as an essential component in all tissues, is the critical parameter involved in the pathogenesis of many diseases (Poole and Arteel, 2016[88]; Sonbol, 2018[106]). ECM is composed of various macromolecules including proteins, glycosaminoglycans, glycoproteins, and growth factors that provide an impactful microenvironment to sustain the viability and functionality of the cells. Furthermore, it is well demonstrated that not only the biochemical composition of ECM, but also its biophysical properties such as stiffness, elasticity, and topography provide essential cues for cell proliferation, differentiation, and migration (Hynes, 2009[50]; Piryaei et al., 2011[86]; Watt and Huck, 2013[128]). Indeed, it is demonstrated that the architecture and biophysical properties of the ECM are critical in disease initiation and progression. Recent studies have highlighted that dynamic changes in matrix stiffness during liver fibrosis progression plays a key role in HSCs activation and changes in the cell-matrix adhesion profile of the liver sinusoidal endothelial cells (LSECs) (Couvelard et al., 1993[17]; Wells, 2005[130]).

## Different Sources of the Cells for in vitro NAFLD Models

For reconstructing human *in vitro* liver models, including NAFLD, three major types of cells are used: primary human hepatocytes (PHHs), hepatocyte-like cells (HLCs), and hepatic cell lines (HCLs). Since each of these cells has its advantages and disadvantages, selecting a suitable cellular source, such as a liver parenchymal cell, is still challenging considering the affordability, availability, reproducibility, and physiological relevance of the generated model.

### Primary human hepatocytes

The application of PHHs is considered the gold standard method for toxicological and pharmacological study. These cells more closely resemble the *in vivo* phenotype as they recapitulate the specific human liver function and metabolism. The harvest of cells from patients suffering from NAFLD has improved the precision of studies on candidate drug metabolism (Ganji et al., 2015[34]; Schwartz et al., 2020[98]). However, the shortage of PHHs and the reliance on patient surgery are the main reasons that using human primary hepatocytes is not very common. Moreover, the donor-dependent variability of PHHs leads to significant variation and low reproducibility in experimental results (Zeilinger et al., 2016[145]; Bell et al., 2018[8]). 

PHHs are terminally differentiated liver parenchymal cells but under *in vitro* conditions show phenotypic instability, limited culture duration, and proliferation. Nonetheless, when PHHs were exposed to the free fatty acids (FFAs) demonstrated an increase in lipid accumulation and endoplasmic reticulum (ER) stress. The steatosis induced by FFAs was significantly reduced after treatment with glucagon-like peptide 1 (GLP-1) (Wobser et al., 2009[135]). This study displays a new role for GLP-1 analog in hindering steatosis progression. Furthermore, Hepatic Stellate cell (HSC) activation was done when these cells were exposed to the conditioned media harvested from FFAs-treated PHH. This finding indicates that lipid accumulation in hepatocytes caused HSC activation, enhanced their apoptosis resistance, and induced the expression of the profibrotic genes including transforming growth factor-beta (TGF-β), and matrix-metalloproteinase-2 (MMP-2), tissue inhibitor of metalloproteinase-1 (TIMP-1), tissue inhibitor of metalloproteinase-2 (TIMP-2), nuclear-factor κB (NF-κB)-dependent monocyte chemoattractant protein-1 (MCP-1) in HSCs. 

In summary, these data display the pathophysiological link between liver steatosis and fibrosis progression. As a result, despite the advantages of PHHs to investigate a range of NAFLD key events, the bottleneck is that these cells are not widely available to generate reproducible *in vitro* NAFLD models for high-throughput assessments, which is essential for commercialization of the approach.

### Hepatocyte-like cells

Recently, hepatocyte-like cells (HLCs) differentiated from human pluripotent stem cells (hPSCs), i.e. embryonic stem cells (ESCs) and induced pluripotent stem cells (iPSCs) (Malik and Rao, 2013[71]). Human ESCs are considered a gold standard and distinguished pluripotent stem cells however, their isolation from human embryos and the possibility of teratoma formation create serious ethical concerns for their use in research and therapy. Human iPSCs are promising alternatives for ESCs derived from somatic cells, particularly the fibroblast, through transcriptional reprogramming. These pluripotent stem cells have the same potential as ESCs and can similarly differentiate into HLCs (Touboul et al., 2010[118]). 

To date, both sources of human PSCs are considered powerful tools in cutting-edge study and very limited in therapy. Many groups have generated HLCs that somehow express liver-specific genes and proteins, secret albumin (ALB), produce urea, and store glycogen, as well as demonstrated Cytochromes P450 (CYPs) enzyme activity and inducibility (Zahmatkesh et al., 2021[142]). However, there is no protocol to produce fully mature hepatocytes which are functionally equivalent to PHHs from these PSCs. Indeed, the HLCs derived from ESC or iPSC, express the immature hepatocyte marker, alpha-fetoprotein (AFP), secrete less ALB, and exhibit significantly decreased CYPs activity compared to PHHs (Bukong et al., 2012[10]; Yi et al., 2012[138]; Schwartz et al., 2014[99]). 

To induce further maturation and metabolic functionality of the HLCs, a variety of approaches, particularly 3D cultivation and co-culture have been used, however, the issue remained (Roy-Chowdhury et al., 2017[92]). Nonetheless, as an alternative to PHHs, these cells can be used for some investigations or limited therapy including patient-derived HLCs generation for drug screening, assessments of inter-individual variability in drug response, or hepatotoxicity (Godoy et al., 2013[37]). Indeed, hiPSC derived from patients with hereditary metabolic diseases can be utilized to model diseases in laboratory conditions. The iPSC technology combination with genome editing technologies, e.g., clustered regularly interspaced short palindromic repeats (CRISPR) and CRISPR-associated protein-9 (Cas9), provides further potential for disease modeling and therapeutic applications (Lyu et al., 2018[69]). 

Holmgren and colleagues (2020[47]) used differentiated iPSCs into the HLCs to generate a NAFLD model in which in response to lipid accumulation and ER stress the inflammatory marker such as tumor necrosis factor α (TNFα) was upregulated. Ouchi et al. (2019[80]) developed an hiPSC-derived model of hepatic steatosis where human pluripotent stem cells are co-differentiated into stromal and epithelial lineages whereupon exposure to FFA shows hepatocyte ballooning, fibrosis phenotype, and an increase in inflammation markers. Despite the advantage of PSCs for disease modeling, so far, there are some limitations to their use due to insufficient functionality, incomplete hepatic differentiation that limits the reproducibility of experiments, and difficulties to produce large volumes of these cells (Ouchi et al., 2019[80]).

### Hepatic cell lines

Human hepatic cell lines are immortalized cells, either derived from liver tumors (e.g., HepaRG, HepG2, Hep3B, SK-Hep-1, and HuH7) or produced through genetically manipulated primary hepatocytes (e.g., SV40 Large T, and hTERT) (Wilkening et al., 2003[134]; Andersson et al., 2012[2]; Samanez et al., 2012[96]). The immortalized cell lines have the wide availability and ability of unlimited proliferation, which makes them more cost-effective compared to PHHs and HLCs and an appropriate cell source for large-scale usages with high reproducibility (Ramboer et al., 2014[89]). 

However, human hepatic cell lines are limited by lower functionality, especially in CYP450 activity compared to PHHs (Guo et al., 2011[42]). Also, some mutations in the immortalized cell lines may significantly alter the cell phenomena compared to normal hepatocytes in the human body. Therefore, the selection of appropriate cell lines for each type of experiment should be considered intelligently. For example, HepG2 cells that are widely used for NAFLD *in vitro* modeling, are homozygous for the PNPLA3 I148M mutation, which is involved in several key metabolic functions (Gunn et al., 2017[41]). 

Huh-7 cells have effective CYP3A4 activity and have been used to study the mechanism of chemicals that improve steatosis and reveal new NAFLD pathogenesis mechanisms (Sivertsson et al., 2010[104]). Khamphaya and colleagues (2018[57]) used the Huh-7 cell line to investigate the mechanism of liver regeneration impairment that occurs in NAFLD. They demonstrated, similar to the rat model and liver biopsy of patients with NAFLD, a decrease in type II inositol 1,4,5-trisphosphate receptor (ITPR2) may account for the impaired liver regeneration in the fat-loaded Huh7 cells. 

HepaRG cells are a human bipotent progenitor cell line able to differentiate into two different types of liver cells, cholangiocyte-like, and hepatocyte-like cells, that may be promising surrogates for PHHs. HepaRG exhibits drug-metabolizing enzymes and drug transporters expression patterns similar to PHHs, so that is becoming an interesting tool for hepatic disease modeling (Lübberstedt et al., 2011[68]; Andersson et al., 2012[2]). 

Tolosa and colleagues (2016[116]) exposed HepaRG cells to 28 compounds that have been reported to induce steatosis for mechanistic studying of drug-induced liver steatosis. Lipid content, ROS production, mitochondrial membrane potential alternation, and lipid metabolism gene expression were assayed to investigate the mechanisms involved in drug-induced steatosis. The finding of this study has clinical relevance because most effects of the drug were 100-fold under the concentrations of the therapeutic plasmatic concentration. These results indicate the value of HepaRG cells as a cell-based assay system for studying the mechanisms involved in drug-induced steatosis. 

Overall, using cell lines for disease modeling facilitates standardized protocols, reproducible, and scalable studies. However, a major drawback in using cell lines is their tumor phenotype and altered metabolic functions which limits true comparison with the human condition.

## Engineering Microenvironment of Hepatic Microtissue

Two main parameters in the cell microenvironment are cell-cell and cell-matrix interactions, which are the key players in tissue development, maintenance, turnover, and functionality, as well as response to regeneration and injury. Therefore, these essential parameters should not be overlooked in the engineering approaches for reconstructing tissue or organ equivalents, including microtissue or organoid generation.

### Cell-cell interactions

The interaction of non-parenchymal cells (NPCs) with hepatoblasts and hepatocytes is critical during liver development, normal physiological state, cellular response to injury, progression of diseases, or regeneration. It has been demonstrated that the addition of NPCs to in vitro cultured hepatocyte systems prevents their dedifferentiation, and improves hepatic function and metabolic response to drug treatment (Hasmall et al., 2000[45]; Wheeler et al., 2014[133]). 

Today various types of primary, stem cell-derived, and immortalized NPCs including liver sinusoidal endothelial cells (LSECs), Kupffer cells (KCs), and hepatic stellate cells (HSCs) are available. Just like PHHs, primary NPCs are isolated from the liver, by enzymatic perfusion (Damm et al., 2013[20]; Pfeiffer et al., 2015[83]; Werner et al., 2015[131]). Due to the high cost and instability of the primary cells, several cell lines or alternative cell types have been used in co-cultures with hepatocytes to provide signals similar to the ones provided by primary NPCs. In this section, we will introduce the proper cell composition for optimizing the microenvironment in liver microtissues for disease modeling.

#### Liver sinusoidal endothelial cells 

LSECs are a unique endothelial cell type in the liver and provide appropriate support to hepatocytes. Even though the presence of LSECs is critical in a healthy liver, they play a critical role in disease initiation and progression. LSECs regulate hepatocyte regeneration and function through hepatic growth factors (HGF) and Wnt2 signaling (Yamane et al., 1994[136]). Moreover, it is demonstrated that paracrine crosstalk between LSECs and HSCs is essential for HSCs activation after liver injury. In this situation, LSECs activate HSCs through the secretion of platelet-derived growth factor (PDGF), fibronectin EIIIA, and exosomes containing sphingosine kinase 1 (SK1) (Jarnagin et al., 1994[52]). 

Moreover, LSECs play a crucial role in the regulation of the liver immune, leukocyte adhesion, and viral clearance (Warren et al., 2007[127]). Since endothelial cells have a critical role in the disease signaling pathway, they are frequently co-cultured with hepatocytes to improve *in vitro* disease modeling (Ding et al., 2010[23]; Natarajan et al., 2017[79]). Ströbel and colleagues (2021[109]) presented a 3D *in vitro* microtissue model by co-culturing of primary LSECs, hepatocytes, Kupffer, and HSCs. Upon exposure to the lipotoxic component, the microtissues show pathophysiological features of NASH, including lipid accumulation and pro-inflammatory cytokines secretion.

Since there is no unique surface marker for LSECs, their identification and isolation are challenging (Poisson et al., 2017[87]). In addition, the primary LSECs are often unstable under *in vitro* culture conditions; therefore, endothelial cells from other sources, such as human umbilical vein endothelial cells (HUVECs), are employed to produce liver microtissues. Co-culture of HUVEC and hepatocyte cells enhanced the hepatocyte-specific function, such as albumin synthesis and CYP450 activity (Takebe et al., 2013[113]; Jin et al., 2018[54]). 

Lasli and colleagues (2019[63]) developed an *in vitro *system by co-culturing HUVECs, and HepG2 cells and demonstrated that in the presence of HUVECs the NAFLD pathogenesis could be more reliably recapitulated (Koui et al., 2017[60]). differentiated iPSCs into liver sinusoid endothelial-like cells (LSELCs). These cells have expressed LSEC genes that are similar to primary LSECs. 

In addition to primary LSECs and LSELCs, some immortalized endothelial cell lines such as human foreskin endothelium (HMEC-1), human adipose microvascular endothelial cells (HAMEC), cord blood-derived endothelial progenitor cells (EPCs), and TMNK-1 have been utilized for hepatocyte co-culture and caused an increase in hepatocyte-specific functions (Chan et al., 2016[12]; Pettinato et al., 2019[82]). 

#### Hepatic stellate cells

HSCs store vitamin A and other lipids in the liver (Bataller and Brenner, 2005[6]) and healthy conditions, are quiescent and constitute 5-8 % of liver cells. After a liver injury, HSCs are activated and transdifferentiated into myofibroblast-like cells and acquire pro-inflammatory and fibrogenic functions. The activated HSCs migrate to the site of the lesion and interact with other cell types to repair the tissue by remodeling the ECM (Sato et al., 2003[97]). Indeed, autocrine and paracrine signals from the microenvironment regulate HSC activation in chronic and acute liver diseases. 

After a liver injury, the apoptotic hepatic cells release damage-associated molecular patterns (DAMPs) and other molecules that result in initiating a signaling cascade in HSCs and synthesis of α-smooth muscle actin (α-SMA), and collagen type I as well as the release of chemokines and cytokines such as connective tissue growth factor (CTGF), transforming growth factor β (TGF-β), macrophage chemoattractant protein 1 (Mcp-1), interleukin-6 (IL-6), C-X-C Motif Chemokine Ligand 1 (CXCL1), and C-C Motif Chemokine Ligand 5 (CCL5) (Fabregat et al., 2016[30]). 

It is well known that co-culture of HSCs with hepatocytes causes to increase in hepatic specific gene expression, including ALB, CYP-450 enzymes, and hepatocyte nuclear factor 4 α (HNF4α), which also improves hepatocytes function such as urea production and albumin secretion (Wei et al., 2018[129]). Taken together, HSCs are critical in liver disease modeling such as NAFLD and fibrosis. 

Feaver and colleagues (2016[31]) developed an* in vitro *NAFLD model by co-culturing primary HSCs, hepatocytes, and macrophages. Upon exposure to lipotoxic stimuli fibrogenic markers, such as TGF-β secretion and extracellular matrix gene expression were increased. However, freshly isolated primary HSCs activate rapidly during cultivation on plastic culture-dish, and their *in vitro* gene expression does not completely replicate that observed *in vivo *(De Minicis et al., 2007[22]; Yin et al., 2013[139]). 

Coll and colleagues (2018[16]) differentiated iPSCs into hepatic stellate like-cells (HSLCs), which were particularly similar to primary HSCs at the transcriptional, cellular, and functional levels. Functional analyses indicated that HSLCs were able to accumulate retinyl esters in lipid droplets and are activated in response to wound-healing mediators, which resemble their counterparts *in vivo. *

Today, several immortalized HSC lines are available for co-culturing, including hTERT-HSC, LI90, GREF-X, TWNT-1, LX-1, and LX-2. LX-2 cell line is widely used for *in vitro* NAFLD modeling. Pingitore and colleagues (2019[85]) generated 3D spheroids composed of HSC (LX-2) and human hepatic cell line (HepG2). They indicated that the compactness of 3D spheroids was facilitated by hepatic stellate cells. These spheroids display lipid accumulation and collagen upregulation upon exposure to FFA. In another study, Huh-7 and LX2 were cultured in different systems, such as monoculture, transwell, and co-culture. The results of this study indicated that the activation of HSCs is independent of lipids accumulation but required cell-cell interaction with hepatocyte cells (Barbero-Becerra et al., 2015[5]).

#### Kupffer cells

KCs are liver-resident macrophages that are involved in host defense as the first innate immune cells and are major cytokine producers in inflammatory responses and liver diseases. KCs in healthy livers have a tolerant phenotype and eliminate toxins from the gastrointestinal tract without an inflammatory response. In diseased livers, KCs show a pro-inflammatory M1 phenotype or an anti-inflammatory M2 phenotype dependent on immune and metabolic microenvironments (Dixon et al., 2013[25]). KCs by M1 function secrete pro-inflammatory cytokines including IFN-γ, tumor necrosis factor-α (TNF-α), IL-1, IL-6, IL-12, IL-23, and reactive oxygen species (ROS); while KCs by M2 function secrete anti-inflammatory cytokines such as PDGF and IL-10 (Bourdi et al., 2002[9]). 

Moreover, KCs induce a variety of cellular responses in the liver microenvironment. LSECs and KCs constitute a scavenging system and make some essential signaling with each other. In such a way, pro-inflammatory cytokines secreted by KCs induce phenotypic changes in LSECs, capillarization, and which cause HSCs to activate (Shetty et al., 2018[102]). Since the KCs can secrete pro and anti-inflammatory factors and are a critical cell type in disease modeling Primary human KCs used in co-culture with PHHs or HCLs for disease modeling (Jiang et al., 2019[53]). 

Suurmond and colleagues (2019[112]) indicated that conditioned media from steatotic spheroids that are composed of HepG2 and HUVECs have the potential to activate primary KCs. Furthermore, they demonstrated that steatotic spheroids incorporating KCs show higher levels of steatotic stages in terms of lipid accumulation and ROS generation. 

However, isolation of KCs from the liver tissue in sufficient numbers is very difficult. Tasnim and colleagues (2019[115]) differentiated iPSCs into functional Kupffer-like cells (KLCs) efficiently, which like primary KCs secreted IL-6 and TNF-α after stimulation. Immortalized KCs have been established in rats and mice, but not in humans. THP-1 is a line of human monocytic cells derived from the peripheral bloodstream of an acute monocytic leukemia patient that could be differentiated into macrophages and are used in co-culture with PHHs or HCLs. Wewering and colleagues (2017[132]) present clear evidence that co-cultures of THP-1 and HepG2 improve in vitro hepatotoxicity testing.

### Cell-matrix interactions

ECM is the main parameter in the hepatic microenvironment and has a crucial role in maintaining hepatocyte functionality and polarization in healthy liver, as well as critical in cellular behavior under disease initiation and progression. It has been demonstrated that in tissue engineering, using an ECM-like scaffold is an insightful approach as it recapitulates the native tissue microenvironment better. To date, a variety of synthetic and natural compounds with different mechanical and biochemical properties have been used to fabricate the artificial 2D substrate or 3D scaffold for liver tissue or organ equivalent construct bioengineering. In this section, we will discuss recreating the appropriate scaffolds/matrices for optimizing the microenvironment in the generation of liver microtissues for disease modeling.

#### Synthetic scaffolds 

Synthetic scaffolds are widely used for tissue engineering, including liver microtissue generation. Unlike natural scaffolds, synthetic scaffolds are more available and fully defined, as well as have tunable properties (Vasanthan et al., 2012[122]). The main disadvantage of synthetic polymers is the low bioactivity caused by the lack of cellular adhesion ligands that can be overcome through chemical modifications. 

Several synthetic polymers, such as polyethylene glycol (PEG), poly-l-lactic acid (PLLA), and Poly lactic-co-glycolic acid (PLGA) have been used to create scaffolds for liver microtissue bioengineering (Linti et al., 2002[67]; Török et al., 2011[117]; Stevens et al., 2015[108]; Grant et al., 2017[40]; Agarwal et al., 2018[1]). PLLA and PLGA are biodegradable polyesters, and their biodegradation rate can be modulated based on the crystallinity of the polymer, and the molecular weight. Both scaffolds are frequently used for hepatocyte cultures, but their success rate is low. 

Wang and colleagues (2013[126]) developed a scaffold composed of a PLLA nanofibrous scaffold coated with type 1 collagen for primary hepatocyte culture and showed superior function in terms of albumin secretion, urea production, as well as CYP1A and uridine 5'-diphospho-glucuronosyltransferase (UGT) enzymatic activity. PEG, a hydrophilic and biocompatible polymer, is commonly used in tissue engineering applications. Embryonic bipotential mouse hepatic cells encapsulated within a PEG hydrogel demonstrated these cells could differentiate into the hepatic lineage, and their gene expressions can be manipulated by siRNA (Underhill et al., 2007[121]). To the best of our knowledge, there haven't been any studies involving a synthetic scaffold for NAFLD modeling. 

#### Natural scaffolds

Recently the use of natural polymers is becoming more and more attractive in tissue engineering because of their biocompatibility and bioactive properties (Vazirzadeh et al., 2022[123]). The most commonly used polymers of natural origin for liver tissue engineering include alginate (Du et al., 2014[27]), chitosan (Tripathi and Melo, 2015[119]), hyaluronic acid (HA) (Katsuda et al., 2010[56]), collagen (da Silva Morais et al., 2020[19]), gelatin (Ruoß et al., 2018[93]), Matrigel (Ryu et al., 2019[94]), and decellularized liver ECM (Saheli et al., 2018[95]). 

Alginate is a commercially available polysaccharide extract found in the cell wall of brown algae, such as genera *Laminaria*, *Macrocystis*, and *Ascophyllum*. Alginate is a hydrophilic polymer and forms a viscous gum after hydration, so it is useful for spheroid generation and promoting cell-cell interactions and hepatocyte functions (Jain et al., 2014[51]). 

Jitraruch and colleagues (2014[55]) used alginate for hepatocytes encapsulation and demonstrated that alginate was able to support human hepatocytes viability after intraperitoneal transplantation of the constructs into a rat with acute liver failure. Miranda and colleagues (2010[75]) encapsulate hepatocyte aggregate in alginate hydrogel and culture in a spinner flask for over one month. This study shows that the use of alginate hydrogels improves the phenotype and functionality of the liver cells. Darakhshan and colleagues (2020[21]) generated microtissues through the co-culturing of Huh-7 cells with mesenchymal stem cells (MSCs) and HUVEC in microcapsules composed of alginate and liver-derived extracellular matrix. The generated human microtissues demonstrated the increased secretion levels of albumin, fibrinogen, alpha-1-antitrypsin, and urea production as well as upregulation of hepatic-specific and drug metabolism-related genes. 

Chitosan is a polysaccharide derived from the cell wall of fungi, crustaceans, algae, microorganisms, insects, and invertebrate animals that are great chitin sources (Agarwal et al., 2018[1]). Various forms of chitosan scaffolds such as hydrogels, microcarriers, membranes, as well micro- and nano-fibers have been used in hepatocyte culture *in vitro*. Due to lack of cellular binding domains and low bioactivity, chitosan is often used with other molecules or functional materials. Wang and colleagues (2016[125]) conjugated lactose moieties to chitosan and improved cellular adhesion and mechanical properties of scaffolds for hepatocytes in culture. Hybrid scaffolds of chitosan with alginate or collagen showed hepatocyte function improvement (Li et al., 2003[66]).

Hyaluronic acid, a glycosaminoglycan of ECM, is the principal component of the perisinusoidal space within the liver and its high hydrophilicity to form a hydrogel makes hyaluronic acid-containing scaffolds appealing for the cultivation of the liver cell. HA can be extracted from rooster combs, bovine cartilage, synovial fluids, umbilical cords, and some microorganisms. Zavan and colleagues (2005[144]) enriched HA with the fibroblast-secreted extracellular matrix and then used it for hepatocyte culture. Hepatocytes cultured on the ECM-enriched HA scaffolds when implanted in nude mice secrete albumin for up to 14 days, generated small and well-organized aggregates, and remain viable up to day 35. Despite the many advantages it has, during liver fibrosis in humans and mice, HA products promote pro-fibrogenic and invasion phenotype of hepatic stellate cells (Yang et al., 2019[137]).

Collagen type I is the most plentiful component of the ECM, which is typically derived from bovine and porcine tissues or rat tail tendons (Soret et al., 2020[107]). Collagen has been used for cell culture in various forms, such as coatings for monolayer 2D cultures, collagen sandwiches, microspheres, and scaffolds for 3D culture systems. The collagen gel sandwich has been traditionally utilized in hepatocytes culture in between double layers of collagen. The resulting structure maintains the native cytoskeletal organization, shows improvements in hepatocyte morphology and polarity, and enhances their liver-specific functions. Collagen has many cell-binding motifs, low antigenicity, and high biocompatibility, but is rarely employed for disease modeling due to low mechanical strength (Dong and Lv, 2016[26]).

Gelatin, a natural compound derived from type I denatured collagen that does not have three alpha-helical structures, is often used as an alternative for collagen. Gelatin is biodegradable and affordable, with high feasibility for 3D scaffold fabrication (Kumari et al., 2016[62]; Ruoß et al., 2018[93]). Hou and Hsu (2020[48]) fabricated a 3D scaffold consisting of chitosan and gelatin with pore structures similar to the liver ECM. They reported the scaffold was able to support hepatocyte viability and functionality *in vitro*. Mohammadpour and colleagues (2018[76]) combined gelatin with laminin to support human adipose-derived mesenchymal stromal cells (ADMSCs) differentiation into hepatocyte-like cells. The ADMSCs seeded on the scaffold showed upregulation of hepatocyte-specific genes and functions. 

Moreover, gelatin could be mixed with methacrylic anhydride for synthesizing gelatin methacryloyl (GelMA) (Zhu et al., 2019[147]). GelMA is bio-compatible and degradable by matrix metalloproteinases (MMPs), which were used to generate 3D lobule-like tissues with hepatocytes (Cui et al., 2019[18]), as well as 3D printed bioink for the culture of adult hepatocytes (Zhuang et al., 2020[148]). Suurmond and colleagues (2019[112]) developed a NAFLD model by encapsulation of HepG2, HUVECs, and KCs in GelMA which displayed NAFLD progression, inflammation, and high levels of cellular stress.

Matrigel is a basement membrane composite material secreted by Engelbreth-Holm-Swarm (EHS) mouse sarcoma cells. The major components of Matrigel are type IV collagen, laminin, entactin, and heparin sulfate (perlecan) (Hughes et al., 2010[49]). Matrigel also contains transforming growth factors (TGF), fibroblast growth factors (FGFs), and enzymes such as MMPs (Talbot and Caperna, 2015[114]). Undefined components, batch-to-batch variations, and the high cost of Matrigel are limitations of its applications. Furthermore, Matrigel does not contain all the essential components of natural ECM to support the cell. Ouchi and colleagues (2019[80]) developed a liver organoid from human PSCs in Matrigel. After FFA exposure, the organoids showed NAFLD phenotype that was characterized by steatosis, hepatocyte ballooning, inflammation, and collagen production.

To provide the most biomimetic microenvironment for *in vitro* hepatocyte culture, decellularized liver ECM has become the most promising candidate as a 2D substrate or 3D scaffold for liver tissue engineering. In this approach, the ECM is isolated from liver tissue by removing the cells. The remaining structure has the same chemical composition and architecture as the native liver, maintaining ECM signaling molecules and improving the expression of genes specific to hepatocytes (Saheli et al., 2018[95]). 

The hydrogels produced from the extracted ECM were recently used for liver organoids and microtissue generation. Saheli and colleagues (2018[95]) indicate that 3D liver-derived ECM hydrogel considerably improves the functional activity of self-organized liver organoids generated via co-culturing Huh-7, HUVEC, and MSC compared to 3D collagen hydrogel. Zahmatkesh and colleagues (2021[142]) showed the incorporation of cell-sized microparticles derived from the liver ECM improves hepatic maturation and functions of HLCs differentiated from hPSCs. In another study using the liver ECM-derived microparticles improved *in vitro* PHH function and gene expression (Heydari et al., 2021[46]). As far as we know, no studies have used the ECM for NAFLD modeling.

### Engineering physiological-like microenvironments

For many years, cell culture has been done in flasks, Petri dishes, and plates, or more recently in bioreactors. In these traditionally produced culture conditions cell-microenvironment interaction, which determines cell function and phenotype and influences cell response to stimuli, is not fully controlled (Sengupta et al., 2014[100]). Lately, the microfluidic approach incorporates various knowledge, including biochemistry, engineering, physics, and biology, for developing a novel technique in cell culture on a micro-scale (Young and Beebe, 2010[140]). 

Over the last decade, microfluidic-based cell culture platforms became more attractive in biological research applications, such as drug toxicity assay, differentiation of the stem cell, and biological processes (Dittrich and Manz, 2006[24]; Wan et al., 2011[124]; Zervantonakis et al., 2011[146]). The main advantage of the microfluidic platform for cell culture includes mimicking *in vivo* cellular microenvironments by precise control of spatial and temporal factors, such as soluble factors gradient, nutrients and oxygen supply, metabolites elimination, and shear stress (Sung et al., 2010[111]; Marimuthu and Kim, 2011[72]; Zervantonakis et al., 2011[146]). Since the *in vivo* hepatocytes' microenvironment is highly perfusing, microfluidic-based cell culture is more appropriate for providing physiologically relevant conditions and preserving the phenotype and functions of the liver cells. However, liver chip throughput is relatively low, which is not appropriate for high throughput and fast industrial applications. 

The integration of biosensors offsets the drawbacks of liver chip devices in terms of throughput. In addition, real-time monitoring rather than endpoint analysis would help to better understand the dynamic changes in disease progression (Asif et al., 2021[4]). Furthermore, organ-on-a-chip-based systems can be engineered to design fine tissue architecture. Moreover, a small volume of microfluidic devices is another advantage of this system. It is demonstrated if hepatocytes culture in a microfluidic channel, even if without flow, it remained functional for 21 days in the presence of sufficient nutrients, due to the accumulation of endogenous growth factors including EGF, HGF, and IGF (Choi et al., 2020[15]). 

In addition, as was previously mentioned in the review, the sourcing of human hepatocytes is associated with significant challenges. So far, there are many robust protocols for hPSCs differentiation into hepatocytes, but these protocols require a combination of expensive appliances and reagents. Furthermore, the resulting hepatocytes exhibit lower levels of enzyme activity compared to adult hepatocytes (Duan et al., 2010[28]). Microfluidic platforms enhance differentiation efficiency and decrease the cost of differentiation protocols by using fewer reagents (Giobbe et al., 2015[36]). 

Moreover, communication between different organs has a critical role in disease progression and drug-induced hepatotoxicity. Therefore, it is necessary to study the interaction between multiple organs and their particular role in the development and progression of the disease. In this regard, connecting a liver chip with other organ chips, which are known as “body-on-a-chip” or “human-on-a-chip” platforms, is emerging as a valuable technology for a better understanding of the complex mechanisms of such disease.

## Bioengineered NAFLD Models

As a high-prevalence disease, NAFLD extremely requires appropriate *in vitro* models to conduct valuable complementary studies for evaluation of the impacts of new treatment strategies (Table 1(Tab. 1); References in Table 1: Barbero-Becerra et al., 2015[5]; Cho et al., 2021[14]; Feaver et al., 2016[31]; Gori et al., 2016[38]; Gurevich et al., 2020[43]; Kizawa et al., 2017[58]; Kostrzewski et al., 2017[59]; Kozyra et al., 2018[61]; Lee and Sung, 2018;[65] McCarron et al., 2021[74]; Ouchi et al., 2019[80]; Pingitore et al., 2019[85]; Slaughter et al., 2021[105]; Suurmond et al., 2019[112]). 

**Figure 2:**
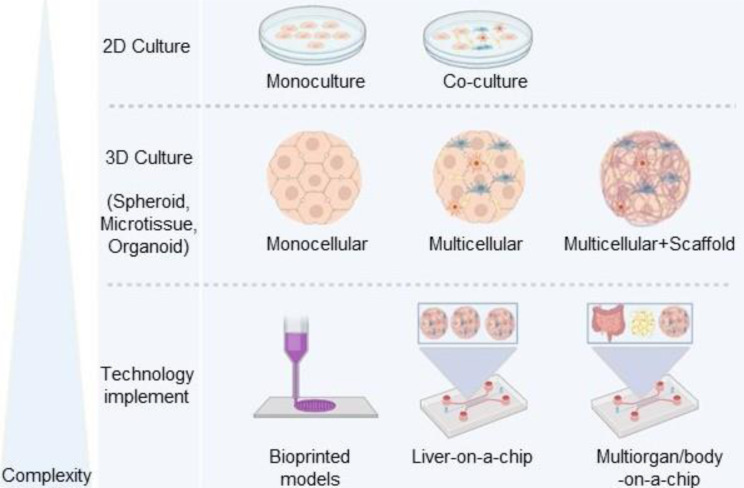
Cell sources, scaffolds, and complexity of *in vitro* models used in NAFLD study. Since from the simple state of the disease (steatosis) to the more advanced stages (NASH and fibrosis), various cell types and tissues involved, therefore more complex *in vitro* models are required. The complexity of the cellular models started from 2D monoculture, 2D co-culture, 3D monoculture, and 3D co-culture scaffold-free or scaffolded, up to organ-on-a-chip, and multiorgan or body-on-a-chip models. In this approach, various sources of parenchymal and non-parenchymal cells such as primary, stem cell-derived, or immortalized cell lines are available to generate the *in vitro* liver models. On the other hand, a variety of synthetic and natural scaffolds or a combination of both of them are optional for biomimetic construct generation. Moreover, microfluidic systems could optimize the *in vitro* condition toward the body's physiology.

Traditionally, throughout exposing the 2D monoculture of PHHs or HCLs to FFA, basic *in vitro* evaluations about NAFLD were achieved. However, reaching multiple intercellular and extracellular networked interactions involved in NAFLD required a 3D co-culture of the liver parenchymal cells with the NPCs, as well as the presence of appropriate substrate/scaffold in a dynamic condition (Figure 2(Fig. 2)). Therefore, in this section, we will have an overview of different *in vitro* models generated for this purpose.

### Monolayer cell culture NAFLD models

Up to now, a lot of studies by the 2D monoculture of PHHs, various human hepatic cell lines (Huh-7, HepG2, HepaRG), and HLCs then exposing these cells to FFA showed some key features in NAFLD, such as steatosis, ER stress, and oxidative stress (Anthérieu et al., 2011[3]; Sharma et al., 2011[101], Graffmann et al., 2016[39]). However, tracking multiple intercellular interactions in NAFLD required the co-culture of these liver parenchymal cells with the other NPCs presented in the tissue. 

Feaver and colleagues (2016[31]) engineered an *in vitro* NAFLD model in a contact-less co-culture fashion in which the PHHs were cultured at the bottom of a transwell whereas human KCs and primary human HSCs were cultured at the top of the transwell. The system integrated hemodynamic-like flow with the media containing elevated FFA, insulin, and glucose levels to induce a NASH phenotype. Their results had some similarities with NASH patients in terms of lipids accumulation, mitochondrial dysfunction, increased oxidative stress, inflammation, and fibrotic markers in the *in vitro* model. However, in this transwell model, the applied non-parenchymal cells have no physical interactions with PHHs.

In another study, a monoculture of LX2 or their co-culture with Huh-7, in both contact-less or contact fashions, were exposed to FFA for 24 hours. FFA induces intracellular lipid accumulation in all three experimental groups, but HSC activation was significantly increased only in contact co-culture conditions (Barbero-Becerra et al., 2015[5]). This result showed that the activation of HSC is independent of the accumulation of FFA but requires cell-to-cell interactions with injured hepatocytes.

### Spheroid-based NAFLD models

Incorporating other liver cell types into NAFLD models can effectively overcome several disadvantages of monoculture systems, but dimensionality and medium flow are still missing. 3D cell culture could better represent the *in vivo* situation, thus proving more pathophysiological or physiological relevance. Also, 3D co-culture systems can be utilized for investigating the more complex features of NASH, such as inflammation and liver fibrosis. 

Kozyra and colleagues (2018[61]) developed a 3D human hepatic spheroid model to mimic steatotic conditions. PHHs from different donors were cultured as a 3D spheroid and supplemented with pathophysiological concentrations of FFA, carbohydrates, and insulin to mimic steatotic conditions and insulin resistance over three weeks. They demonstrated the accumulation of lipids in hepatocytes and the upregulation of lipogenic and insulin-resistant genes. Pingitore and colleagues (2019[85]) generated a model of 3D spheroids consisting of hepatocyte and HSC cell lines (HepG2 and LX-2, respectively). The spheroids showed lipid accumulation and fibrotic features upon exposure to FFA. In addition, they also showed rescued lipid accumulation by treating the spheroids with Elafibranor or Liraglutide which are in clinical trials for NASH treatment. 

In addition to fibrosis, inflammation is also a critical event in NAFLD. Suurmond and colleagues (2019[112]) developed an *in vitro* human liver spheroid for NAFLD by co-culturing HepG2, HUVECs, and Kupffer cells in GelMA and evaluated the disease progression from steatosis to NASH. They demonstrated that upon exposing this microtissue to FFA, KCs could be activated and secrete pro-inflammatory cytokines, as well as higher levels of cellular stress. They concluded that KCs require for creating more reliable *in vitro* human liver models of NAFLD.

### Organoid-based NAFLD models

Derivation of organoids directly from patients with NASH provides a cell source for personalized disease modeling and fundamental NAFLD/NASH biology and facilitates high throughput screening for drug development. 

Ouchi and colleagues (2019[80]) developed a method for a human liver organoid generation where human pluripotent stem cells co-differentiated into epithelial and stromal lineages and then incorporated in matrigel and exposed to retinoic acid in a specific maturation medium. The resulting organoid was composed of multi-cellular including hepatocyte-, stellate-, and Kupffer-like cells whose transcriptome was comparable to native liver tissues. Treatment of these multicellular liver organoids, using increasing doses of FFAs displayed a gradual increase in intracellular lipids accumulation. This organoid recapitulates the stepwise and progressive nature of steatohepatitis and secretes inflammatory cytokines such as IL-6, IL-8, and TNF-α. Prolonged exposure of the organoids with FFAs leads to hepatocyte ballooning as well as upregulation of vimentin and α-SMA. Moreover, upon FFA treatment, the storage rate of vitamin A in the stellate-like cell has declined, indicating that these cells are activated. Additionally, measuring the liver organoid's stiffness by atomic force microscopy (AFM) confirmed the fibrosis.

Gurevich and colleagues (2020[43]) generated liver organoids throughout the co-culture of iPSC-derived hepatocyte-, stellate-, and Kupffer-like cells from NASH donors on collagen-coated plates. They demonstrated that the hepatocytes derived from NASH donors showed accumulation of lipids and recapitulated *in vivo* characteristics, even without FFA supplementation. McCarron and colleagues (2021[74]) generated organoids from the liver cells of patients suffering from NASH and normal livers as controls. The NASH patient-derived organoids exhibited significant up-regulation in their metabolic and pro-inflammatory pathways and showed all typical features of NASH, including reduced albumin secretion, increased apoptosis sensitivity, and lipid accumulation. 

### NAFLD-on-a-chip models 

Due to the capability to mimic *in vivo *physiological parameters, microfluidic-based cell culture improves the metabolic activity and functionality of hepatocytes ensuring that disease progression and drug metabolization are more similar to the *in vivo *condition (Sivaraman et al., 2005[103]). Moreover, the liver is not a lone organ involved in NAFLD, other organs such as adipose tissue and the gut have an important role in the disease initiation and progression. In the past decades, liver-on-a-chip and multiorgan-on-a-chip have been powerful tools for drug screening, liver disease modeling, and studying multiple organ interactions.

Gori and colleagues (2016[38]) developed an advanced microfluidic perfused liver model in which HepG2 was cultured in parallel microchannels that mimic the endothelial-parenchymal interface of a liver sinusoid, allowing the diffusion of nutrients and removal of the wastes similar to the hepatic microvasculature, but actual endothelial cells were not employed in this system. In this microfluidic device, HepG2 cells were perfused in a culture medium supplemented by oleic and palmitic acid, for 24 h and 48 h. They demonstrated a gradual and lower accumulation of intracellular lipids, increased liver cell viability, and minimal oxidative stress in microfluidic dynamic compared with static cultures. Therefore, the chronic condition of in vivo steatosis is more closely replicated in the microfluidic condition. Hence, this system provides a more reliable model than the static 2D cultures for studying NAFLD pathogenesis. 

In addition, Kostrzewski and colleagues (2017[59]) developed a 3D NAFLD perfused platform by using PHH cells. The liver-on-a-chip platform composes of 12 isolated bioreactors in which fluid is recirculated by a micro pump. The channels were coated with a collagen scaffold in which the 3D microtissue could form. After 14 days of exposure to FFA, the hepatocytes showed intracellular lipid accumulation, upregulation of adipokines, and genes associated with NAFLD. Similar to patients suffering from NAFLD, the metabolic activity of the hepatocytes was significantly decreased. On the other hand, when the NAFLD model was treated with anti-steatotic compounds the hepatocytes showed reduced intracellular lipid content compared to untreated controls. However, these platforms focused only on hepatocyte cells, ignoring the importance of their interaction with NPCs, so that unable to reconstruct the suitable liver microenvironment for NAFLD modeling.

Cho and colleagues (2021[14]) developed an advanced liver-on-a-chip platform composed of four cell types, including HepaRG, HUVEC, primary Kupffer cells, and primary stellate cells in GelMA to achieve a human model of hepatic fibrosis derived from NAFLD. They showed accumulation of lipids in hepatocytes (steatosis), neovascularization in endothelial cells, the inflammatory response by activated Kupffer cells (steatohepatitis), and extracellular matrix deposition by activated stellate cells (fibrosis). In this model, the use of stellate cells caused an increase in inflammatory responses and fibrosis markers upregulation. It is suggested that the application of all necessary primary cells or cell lines provide a suitable condition for producing liver microtissue. However, in most differentiation approaches for NAFLD modeling, PSCs only differentiate into HPLCs, and the other essential cell types such as pro-fibrotic and/or inflammatory cells are lacking (Mun et al., 2019[78]).

Lee and Sung (2018[65]) designed a “gut-liver-on-a-chip” platform in which FFAs absorption through the gut layer and then the accumulation of lipids in hepatocytes was demonstrated. This gut-liver chip has two layers that, by a porous membrane, were separated. Gut cells were cultured (Caco-2) on the top layer, and liver cells (HepG2) were cultured on the bottom layer. When butyrate, an anti-steatotic compound that enhances the gut barrier function is administered to the gut compartment, a significant decrease in lipid accumulation in the liver cell is observed. Whereas applying TNF-α by compromising the barrier function of the gut increased hepatic steatosis. This platform had the potential to recapitulate the absorption and lipid accumulation in the gut and liver; moreover, it was a more relevant *in vitro *model of hepatic steatosis than the conventional model of culture. 

Slaughter and colleagues (2021[105]) presented a human-on-a-chip model consisting of human liver and adipose tissue, to evaluate the metabolic factors that contribute to NAFLD initiation and progression. In this study, the indirect effects of adipocytes on hepatocytes upon the progression of NAFLD were validated. 

Gather together, in such novel organ-on-a-chip models, it is possible to interplay between the liver and other organs to address the multi-tissue etiology of NAFLD. However, because of high complexity, high cost, and limitations to scalability, these organ-on-a-chip models were not widely used in laboratories.

### Three-dimensional printed NAFLD models

3D bioprinting could be used to create 3D liver constructs with high accuracy and specific spatial organization. Various bioinks, such as synthetic, natural, and hybrid polymers have been used in 3D bio-printing. Type I collagen has been widely used as a hepatocytes scaffold, but it is less suitable for 3D bio-printing due to its low viscosity and slow rate of polymerization. An improved hybrid of type I collagen and hyaluronic acid yielded a simple, printable bioink that allows PHHs to become viable over two weeks in the resultant construct and respond to drug treatment (Mazzocchi et al., 2018[73]). 

Decellularized liver ECM-derived hydrogel with photo-cross-link capability was created as a bioink to provide specific signals (Ma et al., 2016[70]). However, as far as we know, only one 3D bio-printing approach was used for NAFLD *in vitro* modeling (Kizawa et al., 2017[58]). In this study, cells were collected from genetically obese Zucker rats with NAFLD, and the construction of bio-printed liver tissue was carried out according to the Kenzan methods (Moldovan et al., 2016[77]). On day 23, staining the bio-printed liver structure with Oil-red O showed high lipid content, which indicates that NAFLD conditions can be sustained and monitored in the long term. 

Moreover, 3D bio-printing technology can also improve liver-on-a-chip systems by overcoming limitations in providing various cell types and ECM for spatial heterogeneity (Lee and Cho, 2016[64]). 

## Current Challenges and Future Perspective

In addition to ethical concerns, the major challenge for NAFLD research using animal models is the substantial interspecies differences in the pathophysiology of diseases between animal models and humans, particularly in NAFLD progression and adverse consequences i.e., fibrosis, and cirrhosis. Recent advances in cell culture technology reduced the demand for animal models and provided a wide variety of experimental models that can recapitulate more and more human pathophysiology in the *in vitro* conditions. Although these novel approaches are promising, there are many challenges that should be overcome before making them applicable.

Overall, it appears that co-culture of liver parenchymal and non-parenchymal cells, applying suitable ECM, along with the 3D organization of the generated liver constructs and implementing microfluidic technology are critical parameters that could enhance the physiological relevance of the *in vitro* models. Despite the advantages, these 3D in vitro models are unable to precisely mimic liver-specific architectures, particularly the microvasculature and lobule organization, and more important to recapitulate all pathophysiological events of NAFLD. It could be claiming these 3D culture systems provide only some aspects of native tissue. For instance, bile canaliculi emerged between the hepatocytes in the liver microtissues; however, liver sinusoidal unit with Kupffer cells at their luminal domain and stellate cells at their basal domain were not established (Hammad et al., 2014[44]). Certainly, these cells organization and cooperation are responsible for major mechanisms in toxicity, ranging from interactions with circulating immune cells to pathogenesis of liver fibrosis (Reif, 2014[91]).

Interactions and communication between multiple organs through the blood and lymph circulation as well as endocrine and nervous systems regulatory pathways are essential to deciphering and emulating the temporal processes involved in physiological and pathophysiological functioning of an organ in the human body. For instance, the complex process of absorption/distribution/metabolism/ excretion/toxicity (ADMET), which orchestrates and involves various organs in a precise sequence in which each organ having its own specific function, influence the consequence of taking in an orally consuming toxin through undesirable side-effects in other organs (Cheng et al., 2012[13]; Picollet-D'hahan et al., 2021[84]).

Consequently, to precisely emulate human physiology and pathophysiology, more sophisticated *in vitro* systems should be fabricated via incorporating systemic relations between multiple organs. 3D bioprinted microtissues and organoids will facilitate the generation of intricate structures of the liver models with a distinct spatial organization. Therefore, if the potential advantages of liver-on-a-chip platforms and the 3D construct approach synergistically combine, it will be expected to develop more realistic models of the liver. Additionally, employing such a groundbreaking platform as the multiorgan-on-a-chip with implementing biosensors and real-time monitoring of biochemical parameters can help to deepen our comprehension of the dynamic alterations in molecular and cellular pathophysiology of diseases. 

Even though there have been some advances in multiorgan-on-a-chip research, this field is still in its initial stages. Microfluidics provides the possibility to create intricate physiological scenarios in accurately reproducible *in vitro* conditions. However, the hint for a multiorgan/body-on-a-chip or even an organ-on-a-chip that actually deserves this name has only just begun (Picollet-D'hahan et al., 2021[84]). It is anticipated the next generation of multiorgan-on-a-chip platforms, such as a liver-kidney chip, liver-intestine chip, liver-immune chip, and ultimately, the whole body-on-a-chip system will be developed. These complex systems can open new paths in disease modeling, precision medicine, drug screening, and toxicological studies.

Nevertheless, at the present, fabricating such an advanced *in vitro* model is not only very expensive, time-consuming, and beyond the reach of many researchers, but also has limited scalability to evaluate a high number of chemicals or drugs. Moreover, these high-tech approaches are complicated and necessitate specialized knowledge and proficiency to establish, as well require to be commercialized as cost-efficient products, therefore, it may take a while to be accessible for general usage.

## Notes

Massoud Vosough and Abbas Piryaei (Department of Biology and Anatomical Sciences, School of Medicine, Shahid Beheshti University of Medical Sciences, Tehran, Iran; Tel: +989126971784, E-mail: piryae@sbmu.ac.ir) contributed equally as corresponding author.

## Declaration

### Conflict of interest

The authors declare that they have no conflict of interest.

### Authors' contributions

NA drafted the manuscript and contributed substantially to this manuscript. NR and RG reviewed the draft and prepared the figures. TA critically contributed to reviewing the manuscript. MV and AP developed the concept, contributed to drafting and critical review, and finally approved the manuscript. 

### Acknowledgment

The current article has been written as a part of the comprehensive literature review for conceptualizing a series of research projects on NAFLD modeling which is financially supported by grants received from Royan Institute for Stem Cell Biology and Technology, ACECR (Grant No. 99000166) and Shahid Beheshti University of Medical Sciences (Grant No. 43004196), Tehran, Iran. We would like to express our appreciation to our colleagues at Royan institute, Liver research group, particularly Dr. Zahra Farzaneh for their support. 

## Figures and Tables

**Table 1:**
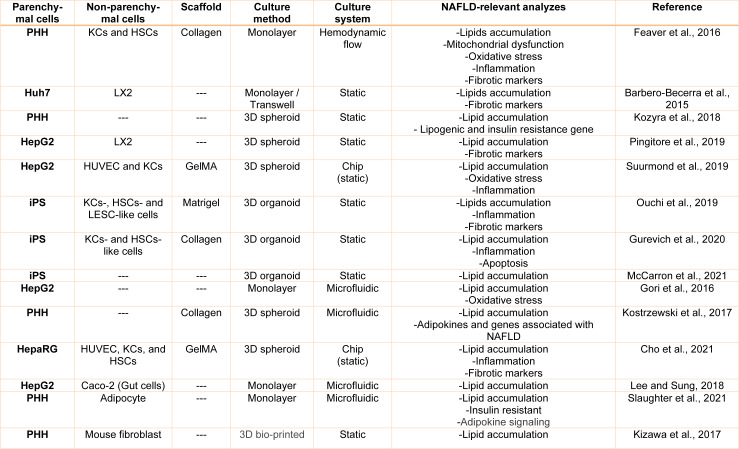
Overview of human in vitro NAFLD Model
